# Protein kinase D2 contributes to TNF-α-induced epithelial mesenchymal transition and invasion *via* the PI3K/GSK-3β/β-catenin pathway in hepatocellular carcinoma

**DOI:** 10.18632/oncotarget.6633

**Published:** 2015-12-16

**Authors:** Yun Zhu, Yang Cheng, YaBin Guo, JinZhang Chen, FengSheng Chen, RongCheng Luo, AiMin Li

**Affiliations:** ^1^ Cancer Center, Traditional Chinese Medicine-Integrated Hospital, Southern Medical University, Guangzhou, Guangdong, China; ^2^ Guangzhou Women and Children's Medical Center, Guangzhou Medical University, Guangzhou, Guangdong, China; ^3^ Liver Tumor Center, Nanfang Hospital, Southern Medical University, Guangzhou, Guangdong, China

**Keywords:** protein kinase D2, hepatocellular carcinoma, epithelial mesenchymal transition, PI3K, β-catenin

## Abstract

Although protein kinase D (PKD) has been shown to contribute to invasion and metastasis in several types of cancer, the role of PKD in the epithelial mesenchymal transition (EMT) of hepatocellular carcinoma (HCC) has remained unclear. We found that PKD2 is up-regulated in HCC and is correlated with the metastasis of HCC. PKD2 positively regulated TNF-α-induced EMT and metastasis of HCC. Mechanistic studies revealed TNF-α-induced PKD2 activation is mediated by the formation of a TNFR1/TRAF2 complex. PKD2 bound directly to the p110α and p85 subunits of PI3K and promoted the PI3K/Akt/GSK-3β signaling cascade to stimulate EMT. In conclusion, our results have uncovered a novel role for the regulation of EMT and suggest inhibition of PKD2 as a potential therapeutic strategy for HCC.

## INTRODUCTION

Hepatocellular carcinoma (HCC) is the sixth most common malignancy worldwide [1]. HCC prognosis is poor due to the high possibility of metastasis and recurrence [2]. It is of critical importance to identify the molecular mechanisms regulating the invasiveness and metastatic potential of HCC.

Protein kinase D (PKD) is a newly described serine/threonine kinase belonging to the calcium/calmodulin-dependent protein kinase superfamily. PKD regulates cell proliferation, survival, migration, and particularly invasion [3, 4]. The PKD family is composed of three members, namely PKD1(PKCμ), PKD2 and PKD3(PKCv), which can be activated by multiple stimuli, such as tumor-promoting phorbol esters, G protein-coupled receptor agonists and growth factors. PKD isoforms are activated *via* the PKC-dependent phosphorylation of a highly conserved serine pair (Ser-744 and Ser-748) in the activation loop, following by being phosphorylated at the auto-phosphorylation sites Ser-916 or Ser-876 [5, 6]. Once activated, PKD isoforms primarily activate pathways including NF-κB, MAPK and CREB to mediate a cascade of reactions [4, 7]. However, the expression and role of PKD isoforms have not been well characterized in HCC.

As a critical proinflammatory cytokine, tumor necrosis factor-alpha (TNF-α) acts as a master switch in establishing the intricate link between hepatitis and HCC [8]. In several cancer cells, TNF-α has been shown to play an important role in the epithelial mesenchymal transition (EMT) [9, 10]. However, whether TNF-α can stimulate EMT and invasion in HCC cells remains unclear.

The primary goal of this study was to investigate the function and regulatory mechanisms of the PKD-mediated signal pathway in TNF-α induced HCC cell EMT and invasion. The relationship between PKD and HCC metastasis was also discussed.

## RESULTS

### PKD2 expression is elevated in HCC

**Figure 1:**
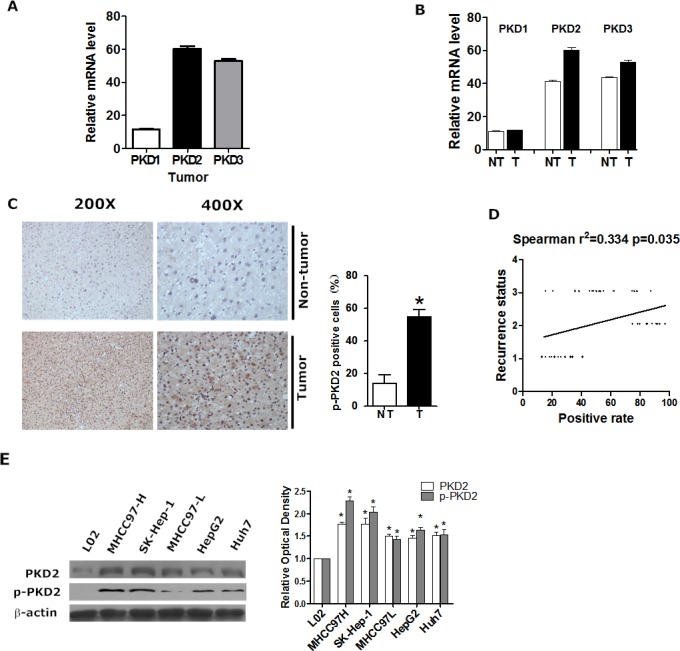
PKD2 is highly expressed in HCC **A.** The relative gene expression levels of PKD1, PKD2 and PKD3 in liver tumor tissues from NCI array database. **B.** PKD1, PKD2 and PKD3 gene expression levels between liver tumor tissues and adjacent non-tumor liver tissues from NCI array database. NT, adjacent non-tumorous liver tissue; T, tumor tissue. **C.** Immunohistochemical staining for p-PKD2 in clinical HCC tissues and normal tissues. Representative photomicrographs (200X and 400X) are shown. The difference in the percentages of p-PKD2-positive cells between normal and tumor tissues was determined by t-test. **D.** The relationship between p-PKD2 in the clinical HCC tissue and recurrence status was estimated using the Spearman method. 1=No recurrence; 2=Recurrence within 3 months; 3=Recurrence after 3 months. **E.** PKD2 and p-PKD2 protein levels were detected by western blot in total protein extracts from all of the cell lines. β-actin was used as a loading control. Data are shown as the mean ± SEM from three independent experiments. *P < 0.05 *vs.* control.

First, we explored the expression of PKD isoforms in human HCC. Analysis of NCI Array database show that, PKD2 was the dominant isoform among all three PKD isoforms expressed in human HCC tissues (*n* = 236) (Figure 1A). Significantly increased mRNA levels of PKD2 were detected in tumor tissues compared with non-tumor tissues (Figure 1B). Also, patients with high predicted metastasis risk signature had higher PKD2 levels compared with those of low predicted metastasis risk signature (Supplementary Figure S1). Analysis of clinical liver tissues was also established. IHC staining for phospho-PKD2 (p-PKD2) was evaluated in 40 liver tumor tissues and 20 non-tumor liver tissues from clinical HCC patients. The percentage of p-PKD2-positively stained cells increased from 18% for normal liver to 57% for liver cancer, and this difference was highly significant (Figure 1C). Moreover, compared with the tumor with no recurrence potential, the expression of p-PKD2 in recurrent tumor was significantly higher, and there was a moderate correlation between the positive rate of p-PKD2 and the recurrence status of the patients (Figure 1D). At the cellular level, the expression of PKD2 and p-PKD2 was significantly higher in HCC cell lines compared with the immortalized human liver cell line L02. Cell lines with high invasive potential (SK-Hep-1 and MHCC97-H) showed higher PKD2 and p-PKD2 expression levels than those with low invasive potential (HepG2, Huh7, Hep3B and MHCC97-L) (Figure 1E). These data suggest that PKD2 is expressed at a high level in both HCC cells and tissues.

### PKD2 contributes to EMT phenotype and invasiveness of HCC

**Figure 2:**
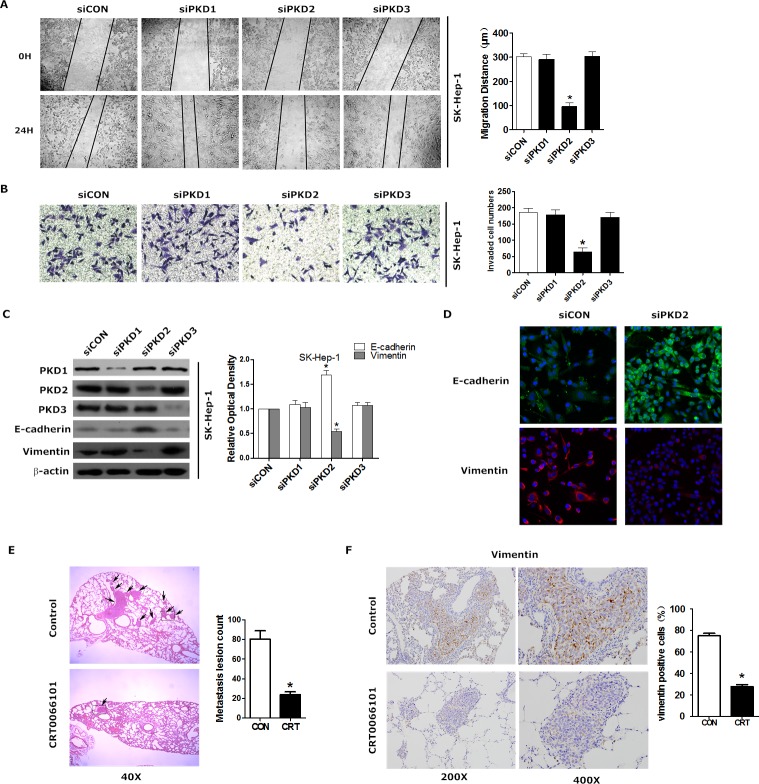
PKD2 contributes to EMT phenotype and invasiveness of HCC **A.** SK-Hep-1 cells were transfected with siCON, siPKD1, siPKD2 or siPKD3 separately and serum-starved for 12 hours. Then, the cells were used for wound healing. Original magnification = 200X. Quantification was carried out by measuring the migrated distance. **B.** SK-Hep-1 cells were treated as described above and seeded onto Matrigel-coated Transwell chambers for 24 hours. Cells that had passed through the membrane were counted. Representative images of cells passing through the membrane are shown. **C.** SK-Hep-1 cells were transfected as indicated. The protein levels of E-cadherin and vimentin were determined by western blot. β-actin was used as a loading control. **D.** SK-Hep-1 cells were transfected with siCON or siPKD2 separately and cultured for 48 hours. Cells were fixed and stained with a primary antibody against E-cadherin and vimentin. Representative photos of confocal microscopy of the samples were shown. **E.** Tumor-bearing mice were treated by gavage with either 5% dextrose (vehicle) or CRT0066101 (80 mg/kg dissolved in 5% dextrose) once daily for one month. Representative HE staining of lungs with tumors was shown. Photographs were taken at magnifications of 40x. Arrows indicate metastatic lesions in the lung. The histologically metastatic lesion number was counted. **F.** Immunohistochemical staining of vimentin was done in liver tissues from mice model. Representative photomicrographs (200X and 400X) were shown. Each experiment was performed in replicate inserts. Data represent the mean ± SEM from three independent experiments. *P < 0.05 *vs.* control.

To explore whether PKD2 affects EMT and invasiveness of HCC, we monitored the change of EMT phenotype as well as the invasiveness by regulating PKD2. Down-regulation of PKD2 led to the suppression of migration (Figure 2A) and invasion (Figure 2B) in the highly metastatic HCC cell line SK-Hep-1. Moreover, down-regulation of PKD2 increased the expression of epithelial markers (E-cadherin and ZO-1) while it decreased the expression of mesenchymal markers (N-cadherin and vimentin) (Figure 2C and 2D). However, the down-regulation of PKD1 and PKD3 has no effect on the migration, invasion or EMT phenotype of HCC cells. This further confirms that it is PKD2, not PKD1 or PKD3 that stimulates EMT and invasion of HCC. A mouse model of pulmonary metastasis was established using SK-Hep-1 to detect the *in vivo* effect of PKD2 inhibitor (CRT0066101) on EMT and invasiveness of HCC. CRT0066101 treatment resulted in a suppression of p-PKD2 in both the primary tumor nodules and the metastasis tumor nodules (Supplementary Figure S2A and B). CRT0066101 also significantly decreased the incidence of pulmonary metastases (Figure 2E). Moreover, the expression of vimentin was significantly inhibited by CRT0066101 in the metastasis nodule in the lung, which consistent with our *in vitro* results (Figure 2F). Collectively, these data suggest that PKD2 promotes EMT and invasion and PKD inhibitor can inhibit EMT and invasion in HCC.

### PKD2 is a key regulator in TNF-α-mediated EMT in HCC cells

**Figure 3:**
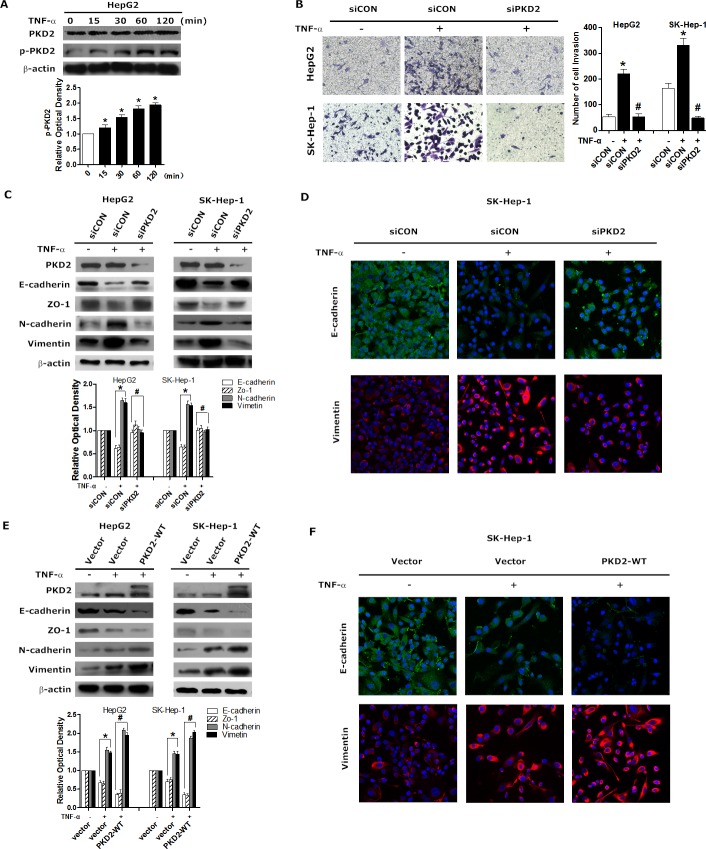
PKD2-mediates TNF-α-induced EMT **A.** Serum-starved HepG2 cells were stimulated with 10 ng/mL of TNF-α for 0, 15, 30, 60 and 120 minutes, and then harvested. The protein level of p-PKD2 was determined by western blot analysis. β-actin was used as a loading control. **B.** HepG2 and Sk-Hep-1 cells were transiently transfected with control or PKD2 siRNA, followed by serum starvation for 12 hours. Then, the cells were seeded onto Matrigel-coated Transwell chambers and treated with solvent or 10 ng/mL TNF-α for an additional 24 hours. Representative images of HepG2 and SK-Hep-1 cells passing through the membrane were shown. **C.** Cells were transfected as indicated. Cells were serum-starved and then treated with solvent or 10 ng/mL TNF-α for 24 hours. EMT markers were analyzed by western blot. **D.** SK-Hep-1 cells were treated as indicated in C. Cells were fixed and stained with a primary antibody against E-cadherin and vimentin. Representative photos of confocal microscopy of the samples were shown. **E.** HepG2 and SK-Hep-1 cells were transiently transfected with GFP-control or GFP-PKD2-WT vectors and then treated with TNF-α or solvent. Western blot analysis was used to detect the expression of epithelial markers and mesenchymal markers. **F.** SK-Hep-1 cells were treated as indicated in E. Cells were fixed and stained with a primary antibody against E-cadherin and vimentin. Representative photos of confocal microscopy of the samples were shown. Experiments were repeated at least 3 times. Data represent the mean ± SEM from three independent experiments. *P < 0.05 *vs*. control. ^#^*P* < 0.05 *vs*. siCON/vector+TNF-α.

Previous reports have identified that TNF-α stimulates EMT in many cancer cells. Consistently, we found that TNF-α treatment markedly increased the motility and invasiveness of both HepG2 and SK-Hep-1 cells (Supplementary Figure S3A and B). TNF-α was also shown to stimulate EMT in a dose- and time-dependent manner (Supplementary Figure S3C). Even more importantly, we observed that p-PKD2 expression gradually increased with time in the presence of TNF-α (Figure 3A), indicating that PKD2 can be activated by TNF-α.

We next examined the alterations of invasiveness and EMT phenotype following the knock down of PKD2 in the present of TNF-α. Transwell assays showed that depletion of PKD2 significantly reduced the number of cells that invaded through the Matrigel in the presence of TNF-α (Figure 3B). Western blot and immunofluorescence showed that depletion of PKD2 rescued the expression of epithelial markers and blocked the up-regulated expression of mesenchymal markers in response to TNF-α (Figure 3C and 3D). We further performed overexpression assays to verify the role of PKD2 in TNF-α induced EMT. Overexpression of PKD2 in HCC cells markedly increased the expression of mesenchymal markers and suppressed the expression of epithelial markers (Figure 3E and 3F). Overall, these results confirm that PKD2 may be crucial for TNF-α-induced EMT in HCC cells. Our study also showed that HCC cells treated by TNF-α developed strengthened anchorage-independent growth ability while knock down of PKD2 inhibited the anchorage-independent growth (Supplementary Figure S4A). IHC assay revealed no correlation between PKD2 and Ki67 or VEGF-A in clinical HCC samples (data not shown). Neither treatment of TNF-α nor depletion of PKD2 can change transcript level of VEGF-A (Supplementary Figure S4B). These results imply that PKD2 may not have effect on proliferation and angiogenesis in HCC.

### TNF-α-induced PKD2 activation is mediated by the formation of a TNFR1/TRAF2 complex

**Figure 4:**
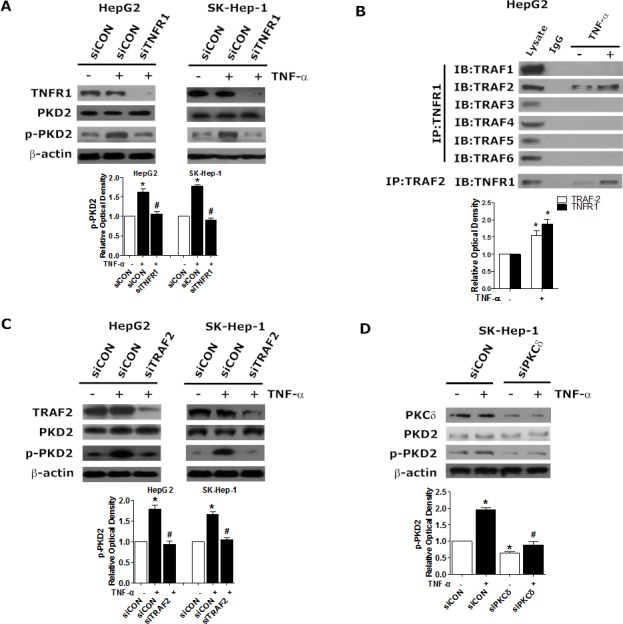
Requirement for TNFR1/TRAF2 in TNF-α-induced PKCδ/PKD2 activation **A.** HepG2 and SK-Hep-1 cells were transfected with TNFR1 siRNA or siCON, serum-starved, and incubated with 10 ng/mL TNF-α or solvent for another 2 hours. The levels of p-PKD2 were analyzed by western blot. **B.** HepG2 Cells were treated as indicated. Cell lysates were subjected to immunoprecipitation using anti-TNFR1 or TRAF2 antibodies. TRAF1-6 and TNFR1 were analyzed separately by western blot. **C.** Cells were transfected with TNFR2 siRNA or siCON in the presence of TNF-α or not and then lysed. The expression of p-PKD2 was detected by western blot. **D.** SK-Hep-1 cells were transfected with PKCδ siRNA or siCON, serum-starved, and incubated with 10 ng/mL TNF-α or solvent for another 2 hours. The levels of p-PKD2 were analyzed by western blot. Experiments were repeated at least 3 times, and representative pictures are shown. *P < 0.05 *vs*. control. ^#^*P* < 0.05 *vs*. siCON+TNF-α.

To investigate whether TNFR1 mediates TNF-α-induced PKD2 phosphorylation, we assessed the effect of modulation of TNFR1 on p-PKD2 expression in HCC cells. As illustrated in Figure 4A, depletion of TNFR1 attenuated TNF-α-dependent high expression of p-PKD2. TNFR associated factor (TRAF) family combine with TNFR1 to mediate TNF-α induced signaling pathways [16, 17]. We further investigated which of the TRAF isoforms participated in activation of PKD2. The presence of TNF-α-induced formation of TNFR1/TRAF complex was revealed by immunoprecipitation. We observed that only TRAF2 can be detected in a TNFR1-immunoprecipitated complex. Immunoprecipitation using a TRAF2 antibody confirmed the interaction between TNFR1 and TRAF2 (Figure 4B). Furthermore, phosphorylation of PKD2 was significantly reduced after transfection with TRAF2 siRNA in HCC cell lines (Figure 4C). As a PKD upstream kinase, PKCδ has been shown to associate with TNF-receptor. We sought to assess the role of PKCδ in TNFα-induced activation of PKD2 by using siPKCδ. We showed that knockdown of PKCδ partly abolished TNFα-induced PKD2 activation in HCC cells (Figure 4D).

Collectively, these data indicate that TNF-α triggers the association between TNFR1 and TRAF2 to induce activation of PKD2 in HCC cells and PKCδ serves as the up-stream of PKD2.

### PKD2 positively regulates GSK-3β/β-catenin pathway in TNF-α induced EMT

**Figure 5:**
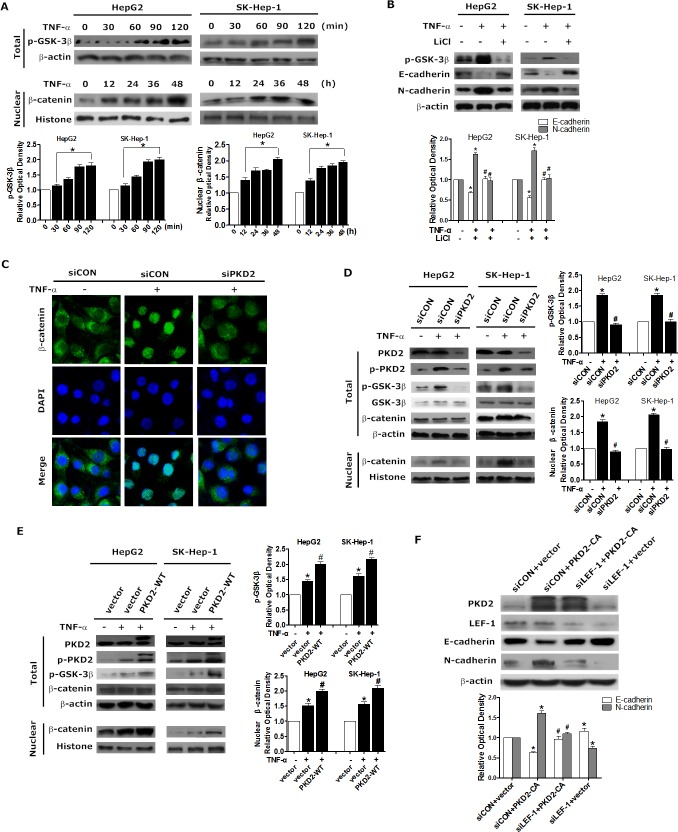
PKD2 regulates GSK-3β/β-catenin pathway in TNF-α-induced EMT **A.** HepG2 and SK-Hep-1 cells were treated with 10 ng/mL TNF-α after 12 hours of starvation. TNF-α treatment, ranging from 0 to 120 minutes, was used to detect changes in p-GSK-3β in the whole cell, and treatment from 0 to 24 hours was used to detect the protein level of β-catenin in the nucleus. Western blotting was used to analyze the levels of p-GSK-3β and β-catenin. β-actin and histone were used as loading controls. **B.** HepG2 and SK-Hep-1 cells were treated with 10 mM LiCl or solvent for 1 hour and 10 ng/mL TNF-α or solvent for another 24 or 1.5 hours. Whole-cell lysates were separated by SDS-PAGE and immunoblotted for p-GSK-3β, E-cadherin and N-cadherin. **C.** HepG2 cells were transfected with PKD2 or control siRNA. 12 hours later, cells were serum-starved and treated with 10 ng/mL TNF-α or solvent for another 24 hours. β-catenin levels were assessed using confocal microscopy. **D.** HepG2 and SK-Hep-1 cells were transiently transfected with PKD2 or control siRNA. 12 hours after transfection, the cells were serum-starved and incubated with 10 ng/mL TNF-α or solvent for 24 or 1.5 hours. Total protein and nuclear protein were extracted seperately. Western blotting was used to analyze the protein levels of total p-GSK-3β and nuclear β-catenin. **E.** HepG2 and SK-Hep-1 cells were transfected with GFP-CON or GFP-PKD2-WT plasmids. After transfection, cells were treated and lysed as described in D. Western blotting was performed to analyze the protein levels. **F.** HepG2 cells were specifically transfected with siCON+vector, siCON+PKD2-CA, siLEF-1+PKD2-CA, siLEF-1+vector. The levels of E-cadherin and N-cadherin were analyzed by western blot. All of the results were confirmed by at least three repetitions, and representative pictures were selected. Data represent mean ±SEM.*P < 0.05 *vs*. control. ^#^*P* < 0.05 *vs*. siCON/vector+TNF-α or siCON+PKD2-CA.

Aberrant activation of GSK-3β/β-catenin pathway leads to enhanced EMT in various cancer cells [18-21]. We therefore examined whether GSK-3β/β-catenin pathway is involved in TNF-α induced invasion and EMT in HCC. In HepG2 and SK-Hep-1 cell lines, the phosphorylation of GSK-3β was elevated in a time-dependent manner in response to TNF-α, as was the level of β-catenin in the nucleus (Figure 5A). The use of LiCl, an effective inhibitor of GSK-3β phosphorylation, dramatically decreased the expression of N-cadherin but elevated E-cadherin level in HCC cell lines (Figure 5B). The results of Transwell assays further showed that LiCl inhibited HCC cell invasion (Supplementary Figure S5). These data suggest that GSK-3β/β-catenin pathway is activated in TNF-α induced EMT in HCC.

Following, we detected the effect of PKD2 on GSK-3β/β-catenin pathway. Depletion of PKD2 led to decreased phosphorylation of GSK-3β as well as the nuclear accumulation of β-catenin in HCC cell lines (Figure 5C and 5D). Meanwhile, increased phosphorylation of GSK-3β was observed in PKD2-overexpressed cells along with the increased nuclear accumulation of β-catenin (Figure 5E). As the transcription factor TCF/LEF1 is under negative control of GSK3β, we measured the effect of depletion of LEF1 on PKD2-overexpressed HCC cells to further confirm the extent of regulation by GSK-3β/β-catenin pathway. Suppression of LEF-1 by siRNA significantly reversed EMT phenotype (Figures 5F). These data together corroborate the positive regulation of PKD2 on GSK-3β/β-catenin signal pathway in TNF-α-induced EMT.

### PKD2 binds to PI3K subunits to stimulate TNF-α induced activation of AKT/GSK-3β

**Figure 6:**
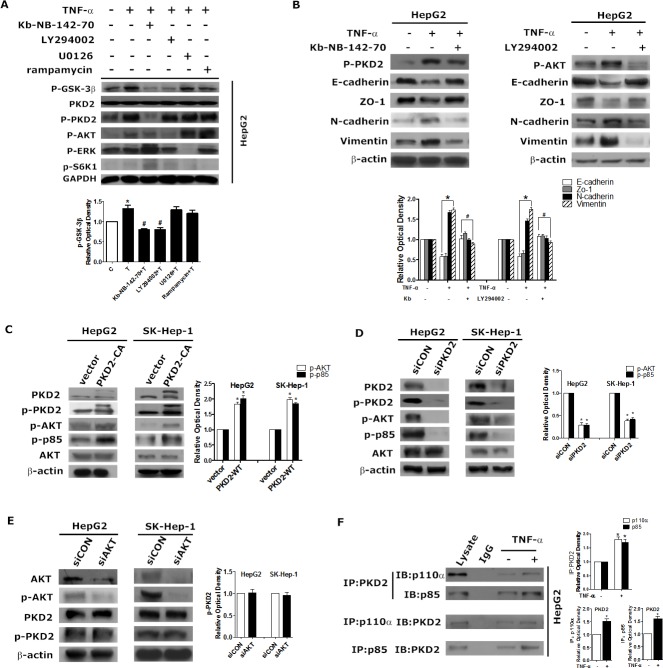
PKD2 regulates the PI3K/AKT pathway in TNF-α-induced EMT **A.** HepG2 cells were treated with solvent, 10 ng/mL TNF-α, 10 ng/mL TNF-α + 5 μM kb-NB-142-70, 10 ng/mL TNF-α + 10 μM LY294002, 10 ng/mL TNF-α + 10 μM U0126 or 10 ng/mL TNF-α + 5 μM rapamycin for 1 hour. Cells in each group were lysed for western blot analysis. GAPDH served as the loading control. **B.** HepG2 cells were serum-starved for 12 hours, pretreated with 5 μM kb-NB-142-70 or 10 μM LY294002 or solvent respectively, and then incubated with 10 ng/mL TNF-α or solvent for 24 hours. **C. D. E.** HepG2 and SK-Hep-1 cells were transfected with GFP-CON/GFP-PKD2-CA plasmids, siCON/siPKD2 or siCON/siAKT respectively (AKT siRNA inhibits AKT1 and AKT2 expression). 48 hours after transfection, total protein was extracted. Western blot was used to detect the indicated proteins. β-actin was used as the loading control. **F.** After treatment with 10 ng/mL TNF-α or medium alone for 30 minutes, whole HepG2 cell lysates were immunoprecipitated with antibodies targeting PKD2. Co-precipitates with p85 and p110α were detected by western blot. After the same treatment, cell lysates were immunoprecipitated with antibodies targeting p85 or p110α, and co-precipitates with PKD2 were detected. Data represent mean value ±SEM of three independent experiments.*P < 0.05 *vs*. control. ^#^*P* < 0.05 *vs*. TNF-α alone.

We explored the precise mechanism by which PKD2 regulates GSK-3β/β-catenin pathway in HCC. As AKT, ERK, 70-kDa ribosomal S6 kinase (S6K1) and PKD have been demonstrated to be upstream regulators of GSK-3β in different cellular contexts [22-25], we thus evaluated which pathways are required for TNF-α induced phosphorylation of GSK-3β in HCC cells. Inhibitors of the above pathways were used. As shown in Figure 6A, among the inhibitors studied, only LY294002 (a PI3K/Akt pathway inhibitor) and Kb-NB-142-70 (a PKD inhibitor) profoundly inhibited the phosphorylation of GSK-3β. Furthermore, as shown in Figure 6B, both LY294002 and Kb-NB-142-70 inhibited the expression of mesenchymal markers while stimulated the expression of epithelial makers in TNF-α induced EMT. These data indicate that PI3K/AKT is a key upstream regulator of GSK-3β in HCC in addition to PKD2.

To explore the underlying association between PKD2 and PI3K/AKT, we assessed the effect of modulation of PKD2 on PI3K/AKT pathway. Overexpression of PKD2 fully activated whereas depletion of endogenous PKD2 impaired phosphorylation of AKT and p85 in HepG2 and SK-Hep-1 cell lines (Figure 6C and 6D). However, the level of PKD2 and p-PKD2 remained stable when endogenous AKT1/2 was depleted by siRNA (Figure 6E). Immunoprecipitation assays showed that two main subunits of PI3K, p110α and p85 directly interacted with PKD2, and this interaction increased in response to TNF-α (Figure 6F). Taken together, these data suggest that PKD2 regulates the PI3K/AKT/GSK-3β pathway by directly binding to PI3K.

## DISCUSSION

The PKD family has been shown to regulate oncogenic progression [4, 14, 26]. The expression patterns and effects of PKD isoforms differ among various types of cancer. PKD1 is reported to play an inhibitory role in cell migration and invasion of several malignancy [27-30]. PKD2 and PKD3 are overexpressed in some highly invasive cancer cells and are demonstrated to accelerate cancer cell invasion through the AKT, ERK and NF-κB signaling pathways [4, 14, 26, 31]. However, the expression and function of PKD isoforms in HCC has never been established. In the current study, PKD2 and p-PKD2 was overexpressed in HCC tissue and HCC cell lines. The level of p-PKD2 was correlated with the recurrence status and the invasiveness of HCC. Animal study demonstrated that inhibition of PKD2 decreased lung metastasis of subcutaneously implanted HCC cells in nude mice. Our findings identify that among the PKD isoforms, PKD2 plays a pivotal role in HCC invasion and metastasis.

HCC progression is a stepwise process accompanied by complex events. EMT is a key event in invasion and metastasis of HCC. We found that PKD2 may act as a key regulator of invasion and metastasis by inducing EMT in HCC cells. This is supported by the fact that ectopic expression of PKD2 promotes, whereas knockdown of PKD2 reverses EMT features, including a change in the expression of epithelial and mesenchymal markers in HCC cells. Anchorage-independent growth is also an *in vitro* marker for a malignant phenotype. We found that PKD2 also played a positive regulatory role in the anchorage independent colony formation of HCC. PKD2 has also been shown to regulate proliferation as well as angiogenesis of tumors [32]. In contrast with these studies, our findings suggest no correlation between PKD2 and proliferation or angiogenesis in HCC.

TNF-α is a proinflammatory cytokine that is highly expressed in HCC [33]. No previous studies have demonstrated TNF-α-dependent induction of EMT in HCC cells. Our study provides evidence that TNF-α induces EMT as well as the migratory and invasive abilities of HCC cells. Furthermore, PKD2 can be activated by TNF-α and the regulation of PKD2 expression resulted in the alteration of epithelial and mesenchymal phenotypes in HCC cell lines, implying that PKD2 may have a positive effect on stimulating TNF-α-induced EMT.

TNF-α activates intracellular signal transduction pathways *via* either of the two membrane receptors, TNFR1 or TNFR2 [34, 35]. Unlike TNFR2, TNFR1 is expressed on almost all cell types, and most of the actions of TNF-α are mediated through TNFR1 [36, 37]. The TRAF family is recruited to the cytoplasmic tails of both TNFRs, and interacts with a variety of proteins to mediate receptor-induced signal transduction [38]. Our study revealed that in HCC cells, TNF-α triggers the association between TNFR1 and TRAF2 which is required for PKD2 activation and the EMT process. PKC is known to phosphorylate the conserved serine residues in the activation loop of PKD and activate PKD. PKCδ, a member of novel PKC, has been shown to associate with TNF-receptor and be involved in the TNF-anti-apoptotic signaling cascade [39]. In accordance with these studies, we found that PKCδ serves as the upstream of PKD2 and is involved in PKD2 activation by TNF-α. This is the first time that the relationship between TNF-α and PKD2 is disclosed in which TNF-α activates PKCδ/PKD2 *via* phosphorylation PKD2 through the TNFR1/TRAF2 pathway.

During tumorigenesis, EMT is associated with aberrant activation of canonical Wnt or the PI3K/AKT pathway, which inactivates GSK-3β and stabilizes β-catenin or Snail, respectively [40, 41]. Our data suggest that PKD2 induces GSK-3β phosphorylation and further induces the translocation of β-catenin into the nucleus to regulate LEF/TCF-dependent transcription to trigger EMT in HCC cells. Moreover, ectopic PKD2 expression increased while the depleted PKD2 expression decreased the phosphorylation of AKT and p85. In addition, we identify a previously unrecognized mechanism for direct regulation of PI3K in HCC. Endogenous PKD2 could directly interact with the p110α and p85 subunits of PI3K to activate PI3K/AKT, which could be enhanced by TNF-α stimulation.

**Figure 7:**
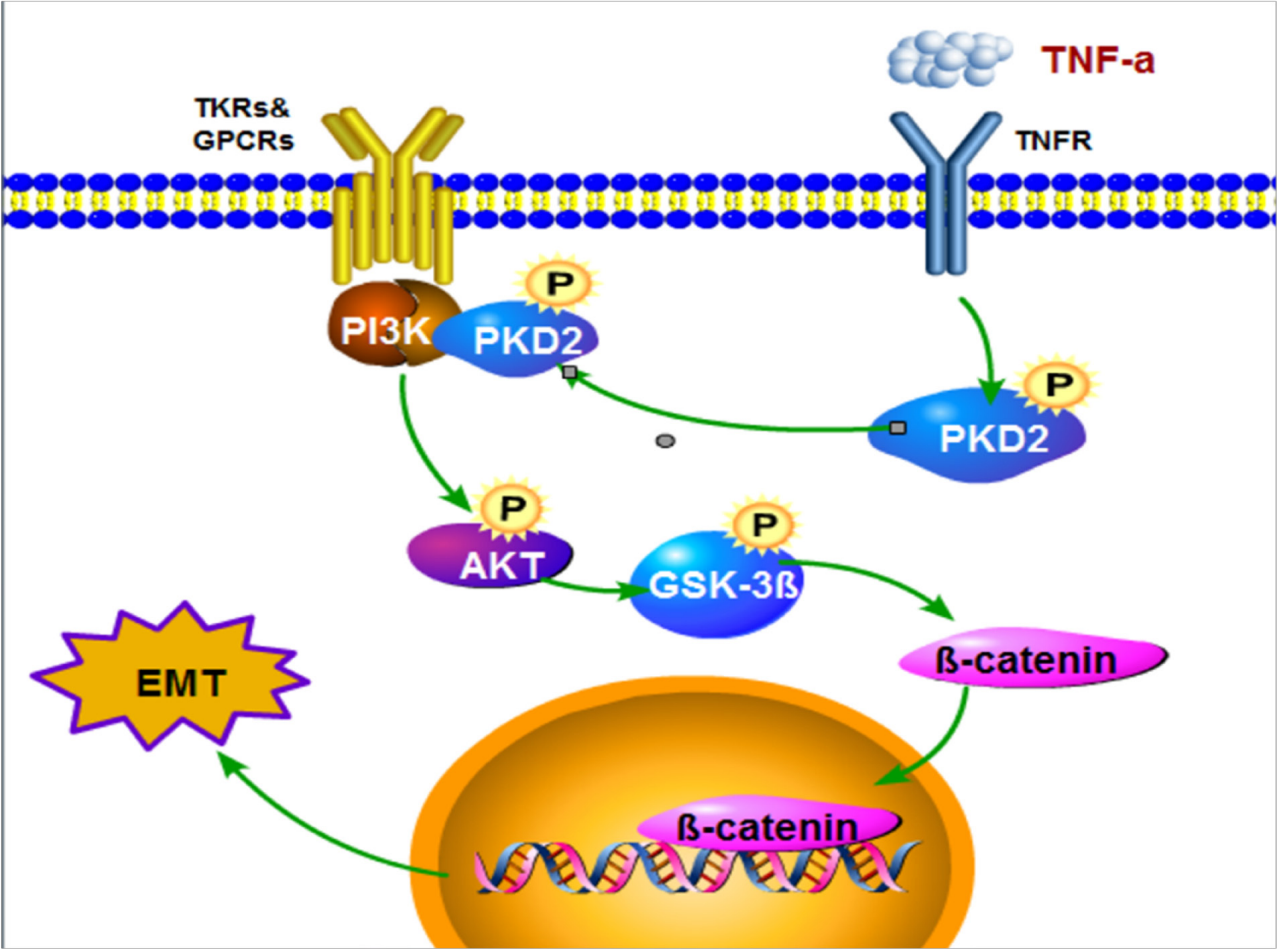
Model describing the proposed role of PKD in the control of TNF-α-induced EMT signaling in HCC TNF-α promotes activation of PKD2 and facilitats the formation of PKD2/PI3K complex via forming TNFR1/TRAF2 complex and thereby accelerates GSK3β/β-catenin pathway.

In summary, this study has shown for the first time that PKD2 regulates EMT and invasiveness of HCC and the expression of PKD2 is related to the metastasis and recurrence potential of HCC. Our findings identify a previously unrecognized mechanism for PKD2 regulated EMT. PKD2 directly associates with p110α and p85 subunits of PI3K. The constitutive association of PKD2/PI3K complex enhanced by TNF-α increases activity of AKT/GSK-3β/β-catenin and promotes EMT and invasiveness of HCC (Figure 7). Our study provides a new potential strategy for HCC therapy.

## MATERIALS AND METHODS

### Chemicals and antibodies

Recombinant human TNF-α was obtained from Cayman (Ann Arbor, MI, USA). Lipofectamine 2000 and Trizol reagents were purchased from Invitrogen (Carlsbad, CA, USA). The nucleoprotein and cytoplasm-protein extraction kits and ready-to-use immunohistochemical SP kits were purchased from KeyGEN BioTECH (Nanjing, JiangSu, China). The SignalStain DAB substrate kit was purchased from Cell Signaling Technology (Danvers, MA, USA). Antibodies targeting AKT, β-catenin and all unconjugated secondary antibodies were purchased from Santa Cruz Biotechnology (Santa Cruz, CA, USA). Antibodies targeting phospho-AKT (p-AKT) and E-cadherin were purchased from Epitomics (Burlingame, CA, USA). Anti-phospho-PKD2 antibodies (for the IHC Assay) were purchased from Sigma. Antibodies against N-cadherin, vimentin, Zo-1, phospho-GSK3β (p-GSK3β), PKD1, PKD2, PKD3, phospho-S6K1(p-S6K1), phospho-ERK1/2 (p-ERK1/2), β-actin and histones were obtained from Cell Signaling Technology. Antibodies targeting green fluorescent protein (GFP) were purchased from Abcam (Cambridge, MA, USA). Alexa-488- and 594-conjugated secondary antibodies were purchased from Molecular Probes (Invitrogen).

### Plasmids and RNA interference

GFP-PKD2 vectors were kindly provided by Dr. Yasuharu Nishimura and Dr. Atsushi Irie (Kumamoto University, Japan). Negative control siRNAs, as well as PKD2 siRNA, Akt siRNA, TNFR1 siRNA, TRAF2 siRNA, GSK-3β siRNA and PKCδ siRNA were received from Cell Signaling Technology.

### Cell culture, drug treatment and transfections

HepG2, SK-Hep-1, L02, MHCC97-H, MHCC97-L and Huh7 cells were obtained from the Oncology Laboratory of Southern Medical University. Cells were cultured in high-glucose DMEM (Life Technologies Corporation, Carlsbad, CA, USA) and supplemented with 10% FBS in a humidified atmosphere of 5% CO2. Cells at 70-80% confluence were maintained in serum-free DMEM for 12 hours before stimulation with TNF-α. Inhibitors were given 30 minutes before TNF-α treatment, if needed. For transient transfection, cells were transfected with plasmid or siRNA using Lipofectamine 2000 reagent, according to the manufacturer's instructions.

### Immunoprecipitation and western blot analysis

Total protein and nuclear protein was extracted according to the manufacturer's protocol. The protein concentration was quantified using the BCA protein assay. The immunoprecipitation was carried out as described before [11], and the precipitated proteins were analyzed by western blotting, which was performed as previously described [12]. The western blot data was quantitated using densitometry. All experiments were repeated at least 3 times.

### Transwell assay

The upper chamber of the Transwell inserts was coated with 50 μL of 2.0 mg/mL Matrigel (BD Biosciences, Franklin Lakes, NJ, USA) before use. 5 × 10^4^ cells, cultured in 200 μL DMEM, were seeded in the upper chamber of the Transwell insert, while 600 μL medium with 10% FBS was used in the lower chamber. Cells were cultured for 24 hours. All cells that invaded to the underside of the membrane were fixed and dyed using crystal violet. Six random fields of view were selected to count the cells, and the mean value was calculated from three independent experiments.

### Wound-healing assay

Cells were seeded onto 24-well plates and grown to confluence. After serum starvation in medium for 12 hours, cells were gently wounded using a pipette tip. Wounded cells were immediately treated as indicated. Images were taken immediately after wounding and after 24 hours. The motility of cells was assessed by the distance between the wound edges.

### Soft agar assay

1×10^4^ pre-treated HCC cells were plated in growth medium containing 0.4% agarose in six-well plates onto a bottom layer of solidified medium containing 0.8% agarose. After incubation for 14 days, colonies were stained with crystal violet solution, washed repeatedly with distilled water. Viable colonies larger than 0.1 mm were counted.

### Immunofluorescence microscopy

After treatment, cells were fixed in methanol for 15 minutes and permeabilized with 0.2% Triton X-100 for 5 minutes at room temperature and rinsed in PBS with 3% bovine serum albumin. After blocking in PBST buffer for 1 hour at room temperature, cells were incubated with antibodies specific for β-catenin, E-cadherin and vimentin overnight at 4°C. Cells were then rinsed with PBS and subsequently incubated with Alexa-488- or Alexa-594-conjugated secondary antibodies for 1 hour at room temperature. The slides were mounted in ProLong Gold Antifade Reagent with DAPI (Invitrogen) and imaged with a Nikon Eclipse E400 fluorescent microscope.

### Microarray dataset analysis

A study involving 236 HCC patients, representing the largest number of subjects reported in the database were selected from NCI caArray database [13]. The expression levels of PKD1, PKD2 and PKD3 in tumor tissue were determined and compared. Then, PKD2 and PKD3 expression in HCC and non-tumoral liver tissues was compared. The correlation between the expression of PKD2 and predicted metastasis risk was also determined.

### Tissue samples and immunohistochemistry

Samples from HCC patients from Nanfang Hospital were confirmed by pathology. Specimens from 40 samples of liver tumor tissue and 20 samples of adjacent normal tissue were collected. Patients’ characteristics and histological data are shown in Table 1. The immunohistochemistry (IHC) and hematoxylin and eosin (HE) staining were performed as described before [14], antibody against phospho-PKD2 (p-PKD2), PKD2, Ki67 and VEGF-A was used. Images were captured using ImagePro software. Two experienced researchers counted the immune-positive cells in a blinded manner.

**Table 1:** Characteristics of patients with HCC

Characteristic	No. of patients	Percentage
Gender		
Male	29	72.5
Female	11	27.5
Age		
≤ 45	25	62.5
>45	15	37.5
Histological classification		
High differentiated	10	25
Moderately differentiated	22	55
Low differentiated	8	20
TNM		
I-II	26	65
III-IV	14	35
AFP		
≤ 400	16	40
> 400	24	60
Tumor size		
≤5cm	25	62.5
>5cm	15	37.5
Recrudescence status		
recrudescene	28	70
no-recrudescence	12	30

### Real-time quantitative RT-PCR

Total RNA was extracted from treated HCC cell lines with Trizol reagent following the protocol. The mRNA was reverse-transcribed from 500 ng of total RNA with Prime Script RT reagent kit (Takara Bio Inc.). Quantitative real-time RT-PCR of VEGF-A was carried out by specific primers using Thermal Cycler Dice Real Time (Takara Bio Inc.) according to the protocol. The primers used for quantitative PCR are listed in Supplementary Table S1.

### Animal experiments using a metastasis mouse model of human liver cancer

Animal research has been approved by Ethics Committee of Southern Medical University. A nude mouse model of lung metastasis of human liver cancer was established as previously described[15]. Tumor-bearing mice were randomly split into CON and CRT groups (*n* = 6) and were treated by gavage with either 5% dextrose (vehicle) or CRT0066101 (80 mg/kg dissolved in 5% dextrose) once daily. 4 weeks later, the mice were euthanized. Subcutaneous tumors were harvested for western blot analysis. Consecutive tissue sections were made for the lung and stained with HE. The number of lung metastatic nodules was counted under the microscope. The specimens of liver were immuno-stained using antibodies to p-PKD2 and vimentin.

### Statistical analysis

All data, expressed as mean±standard error of measurement, were from at least triplicate experiments. The unpaired Students t-test and analysis of variance (ANOVA) were used for statistical analysis. All statistical analysis was conducted using GraphPad Prism 5 software. A P-value less than 0.05 was considered statistically significant.

## SUPPLEMENTARY MATERIAL FIGURES AND TABLE


