# Supplementary material for: Protein kinase D2 contributes to TNF-α-induced epithelial mesenchymal transition and invasion via the PI3K/GSK-3β/β-catenin pathway in hepatocellular carcinoma
Source: Oncotarget. 2015 Dec 16;7(5):5327–41. doi: 10.18632/oncotarget.6633 (PMC4868689; doi:10.18632/oncotarget.6633)
Supplement: Supplementary file 1 [file oncotarget-07-5327-s001.pdf]

Protein kinase D2 contributes to TNF-α-induced epithelial mesenchymal transition and invasion *via* the PI3K/GSK-3β/β-catenin pathway in hepatocellular carcinoma

Supplementary Material

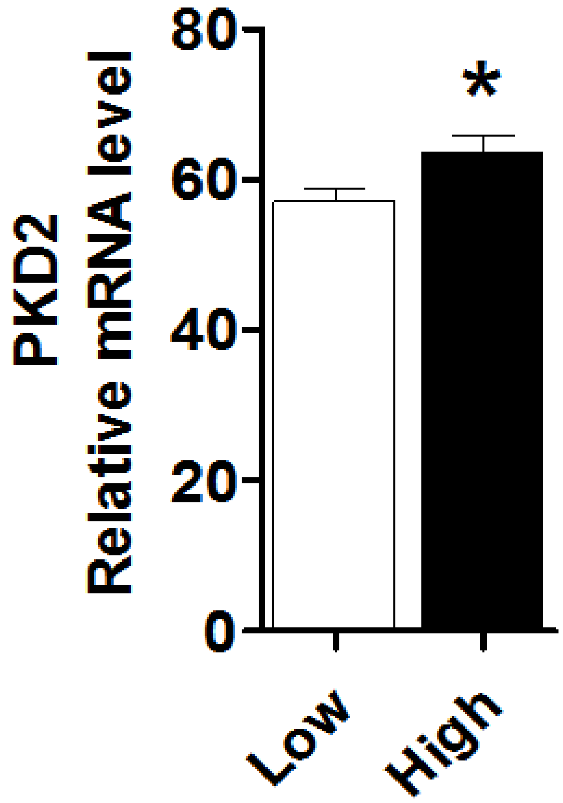

Figure S1: PKD2 gene expression in NCI array database. PKD2 gene expression levels in liver tumor tissues from patients with high or low predicted metastasis risk signatures. Low: Low predicted metastasis risk signature. High: High predicted metastasis risk signature. * $P<0.05$ versus the Low group.

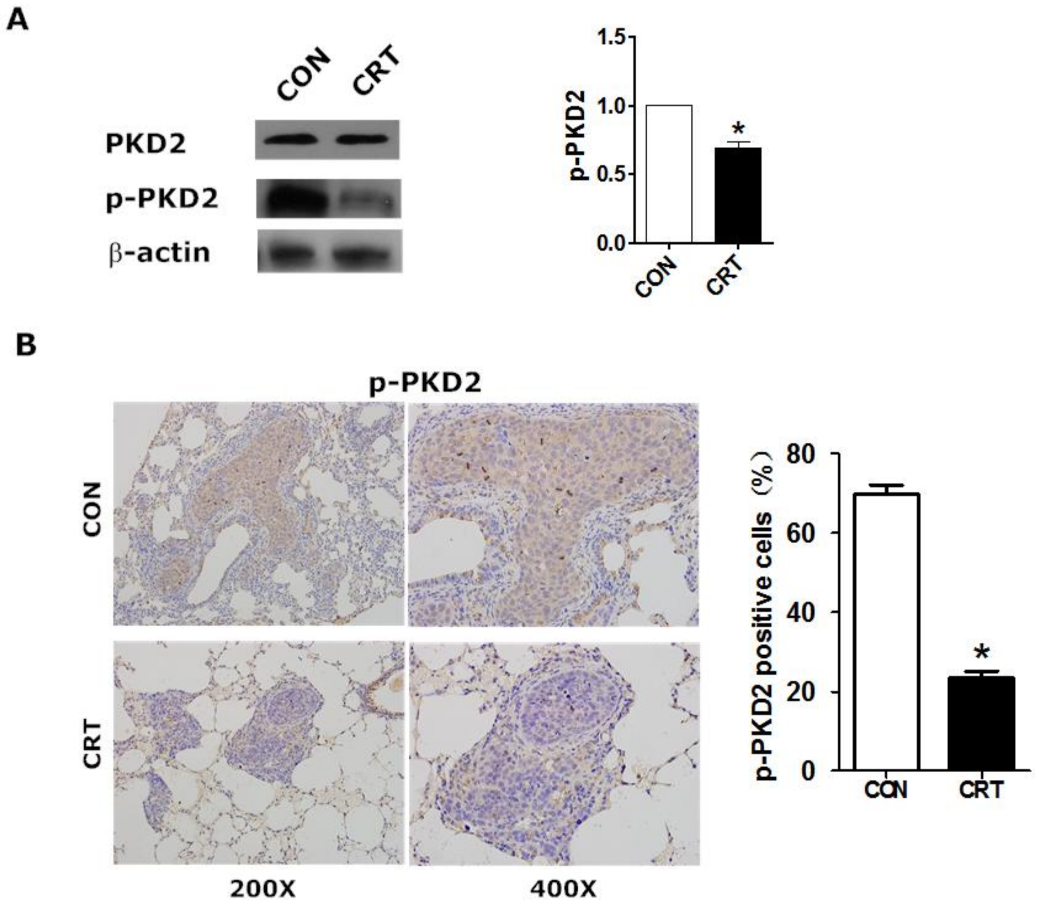

Figure S2: PKD2 inhibitor inhibits the expression of p-PKD2 in a mice model of HCC. A. The expression of p-PKD2 in the primary tumor tissue from mice model was examined by western blot. β-actin was used as a loading control. Data are represented as the mean ± SEM of three independent experiments. **B.** The expression of p-PKD2 in the lung nodules from mice model was examined by IHC. Representative photomicrographs(200X and 400X) are shown. *P<0.05 vs. the control group.

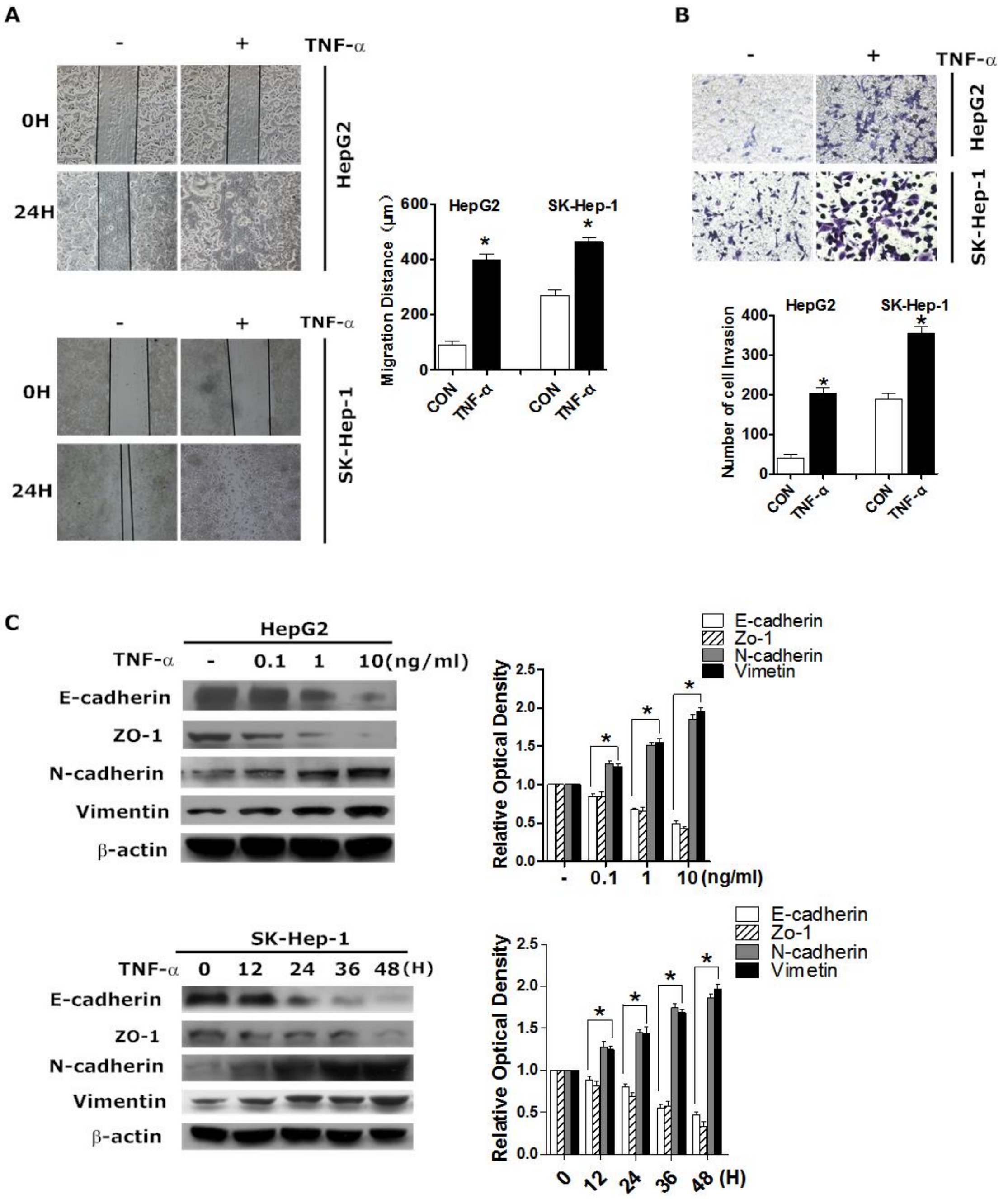

Figure S3: TNF-α induces EMT and invasion in HCC cell lines. A. HepG2 and SK-Hep-1 cells were treated with 10 ng/mL TNF-α or equivalent solvent. Wound healing at 0 hours and 24 hours was observed using an inverted microscope. Quantification was carried out by measuring the migrated distance. **B.** Transwell assays were performed in HepG2 and SK-Hep-1 cells that were treated as above. After twenty-four-hours, images of cells that had

passed through the membrane were taken using an inverted microscope. Six visual fields were randomly selected for counting. Data are represented as the mean ± SEM from three independent experiments. Original magnification = 200X. **C.** HepG2 cells were serum-starved for 12 hours and stimulated with TNF-α (0, 0.1, 1, and 10 ng/mL) for 24 hours. SK-Hep-1 cells were serum-starved for 12 hours, followed by stimulation with 10 ng/mL of TNF-α for 0, 12, 24, 36 and 48 hours. Western blot was used to detect the indicated proteins. β-actin was used as a loading control. Data are represented as the mean ± SEM of three independent experiments.* $P<0.05$ vs. the control group.

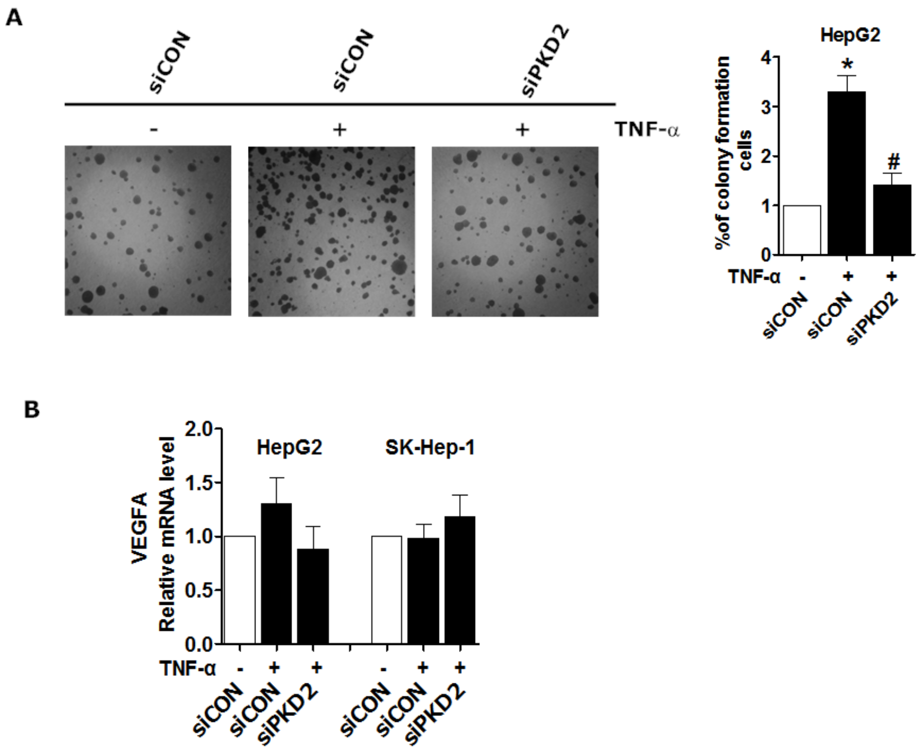

Figure S4: The role of PKD2 in anchorage-independent growth and angiogenesis of HCC. A. HepG2 cells transfected with siPKD2 or the control siRNA were planted in soft agar and grown for 14 days in the presence of TNF-α (10 ng/mL) or solvent and stained with crystal violet solution. Viable colonies larger than 0.1 mm were counted. Images are representative. The values are expressed as percentage of colonies relative to siCON-transfected cells. **B.** HepG2 and SK-Hep-1 cells were transfected with PKD2 siRNA or siCON, serum-starved, and incubated with 10 ng/mL TNF-α or solvent for another 2 hours. Total RNA was isolated. The mRNA level of VEGF-A was examined by real-time qPCR analysis. GAPDH was used as a loading control. Data are represented as the mean ± SEM of three independent experiments. * $P<0.05$ vs. the control group. $^{\#}$ $P<0.05$ vs. TNF-α alone.

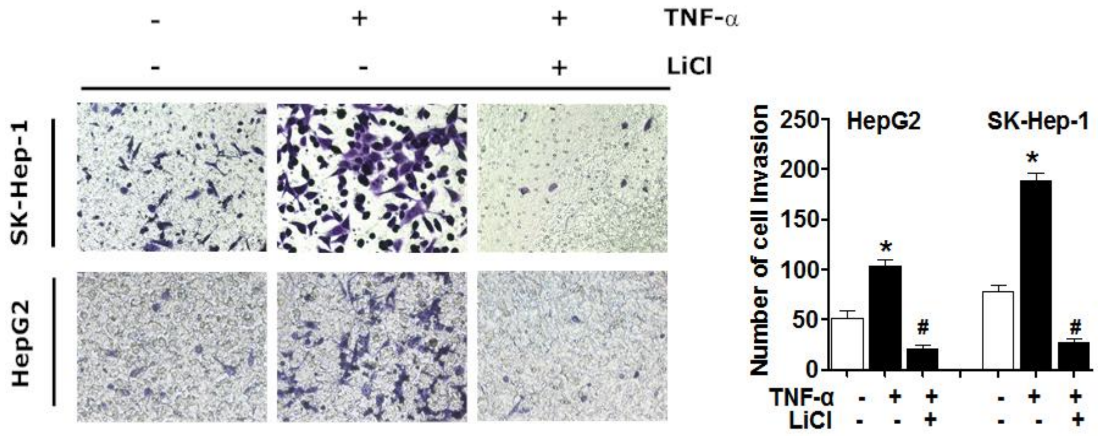

Figure S5: GSK3β inhibitor inhibits invasiveness of HCC. Transwell assays were performed in HepG2 and SK-Hep-1 cells after treating with 10 mM LiCl or solvent for 1 hour and 10 ng/mL TNF-α or solvent for another 24 hours. Cells that had passed through the membrane were counted. Representative images are shown. Data represent the mean ± SEM for three independent experiments. * $P<0.05$ vs. control. # $P<0.05$ vs. TNF-α alone.

Table S1. Sequence of primers for RT-PCR and qRT-PCR

Gene	Forward primer	Reverse primer
VEGF-A	5′-CCCTGATGAGATCGAGTACATCT-3′	5′-GCCTCGGCTTGTCACATTTT-3′
GAPDH	5′-TGTTCGTCATGGGTGTGAAC-3′	5′-ATGGCATGGACTGTGGTCAT-3′
